# Genome-wide analysis of auxin transport genes identifies the hormone responsive patterns associated with leafy head formation in Chinese cabbage

**DOI:** 10.1038/srep42229

**Published:** 2017-02-07

**Authors:** Li-wei Gao, Shan-wu Lyu, Jun Tang, Dao-yun Zhou, Guusje Bonnema, Dong Xiao, Xi-lin Hou, Chang-wei Zhang

**Affiliations:** 1 State Key Laboratory of Crop Genetics and Germplasm Enhancement/Key Laboratory of Biology and Germplasm Enhancement of Horticulture Crops in East China, Ministry of Agriculture, Nanjing Agricultural University, Nanjing, 210095, PR China; 2 Institute of Horticulture, Jiangsu Academy of Agricultural Sciences, Nanjing, PR China; 3 Wageningen UR Plant Breeding, Wageningen University and Research Centre, Wageningen, the Netherlands

## Abstract

Auxin resistant 1/like aux1 (*AUX*/*LAX*), pin-formed (*PIN*) and ATP binding cassette subfamily B (*ABCB*/*MDR*/*PGP*) are three families of auxin transport genes. The development-related functions of the influx and efflux carriers have been well studied and characterized in model plants. However, there is scant information regarding the functions of auxin genes in Chinese cabbage and the responses of exogenous polar auxin transport inhibitors (PATIs). We conducted a whole-genome annotation and a bioinformatics analysis of *BrAUX*/*LAX, BrPIN*, and *BrPGP* genes in Chinese cabbage. By analyzing the expression patterns at several developmental stages in the formation of heading leaves, we found that most auxin-associate genes were expressed throughout the entire process of leafy head formation, suggesting that these genes played important roles in the development of heads. UPLC was used to detect the distinct and uneven distribution of auxin in various segments of the leafy head and in response to PATI treatment, indicated that the formation of the leafy head depends on polar auxin transport and the uneven distribution of auxin in leaves. This study provides new insight into auxin polar transporters and the possible roles of the *BrLAX, BrPIN* and *BrPGP* genes in leafy head formation in Chinese cabbage.

Chinese cabbage (*Brassica rapa L.* ssp. *pekinensis*), which originated in China, is one of the most important *Brassica* vegetables. As the nutrient storage organ, the leafy head is composed of incurved, yellowish leaves, with good taste and abundant nutrients, including multiple types of dietary fiber and vitamins, all of which are important parameters of leafy head quality. Poor heading of leaves can often cause a considerable yield reduction^1^. The economic benefits of a leafy head depend on many factors, such as temperature, light intensity, auxin concentration, and the carbohydrate-to-nitrogen ratio. Among these factors, auxin is the most important, and an uneven distribution of auxin concentration in Chinese cabbage can modify the characteristics of head formation. However, the molecular mechanism and genetic basis of leafy head development remain poorly understood.

It is commonly accepted that auxin is necessary for the growth and development of plants^2^,^3^,^4^ and is the only plant hormone with the feature of polar transport, which can determine the plant morphology^5^. Indole-3-acetic acid (IAA) is the principal auxin^6^,^7^,^8^, and its concentration and uneven distribution in leaves can modify the apical dominance, the tropic growth, the senescence delay, and the differentiation of xylem and phloem; therefore, polar auxin transport (PAT) is the primary decision-maker of plant morphology^5^,^9^,^10^,^11^. There is some evidence showing that leafy head formation in Chinese cabbage may depend on a relatively high auxin concentration and uneven auxin distribution in head leaves^1^. After the rosette stage, folding leaves began to bend inward and upward under the effect of an auxin gradient. The leaves of Chinese cabbage curve inward as they are exposed to higher auxin content.

Recent results indicated that auxin is biosynthesized in the meristematic tissue regions at the shoot apex and forms auxin gradients that regulate cellular events by coordinating the actions of influx (*AUX*/*LAX*), efflux PIN-FORMED (*PIN*) and ATP binding cassette subfamily B (*ABCB*/*MDR*/*PGP*) carriers in the plasma membrane^5^,^12^,^13^,^14^,^15^. The *AUX*/*LAX* gene family is composed of four members, *AUX1, LAX1, LAX2* and *LAX3*^16^. In a previous study, *AUX1* mutants of *Arabidopsis* were used to investigate the function of the auxin influx carrier^17^. *AUX1* and *LAX1* are two regulators of phyllotactic patterning in *Arabidopsis,* and *LAX2* has been reported to regulate vascular patterning in cotyledons. *LAX3* has been reported to participate in lateral root emergence and to facilitate auxin uptake^16^,^18^. The auxin efflux carrier *PIN* genes were the first to be identified as essential for PAT^15^,^19^,^20^. The *PIN* gene family, a small multigene family, consists of eight members called *AtPIN1* to *AtPIN8*. Previous research showed that *PIN*s are involved in various biological processes in plant development, including mediating the auxin concentration and playing a role in gravity-sensing tissues, as well as regulating pollen development and function and intracellular auxin transport^15^,^21^,^22^,^23^,^24^. The resistance-type ATP binding cassette subfamily B (*ABCB*) possesses 21 transcribed genes and 1 pseudogene in *Arabidopsis*. A previous study suggested that *PGP* genes played a critical role in cellular and long-distance auxin transport^25^. Among the *AtPGP* family, at least six members (*PGP1, 4, 14, 15, 19*, and *21*) mediate the process of auxin update in *Arabidopsis*^25^,^26^,^27^,^28^.

Recently, PAT has been demonstrated by auxin efflux inhibitors, such as 2,3,5-triiodobenzoic acid (TIBA) and 1-N-naphthylpthalamic acid (NPA)^29^. These two PATIs were used to identify the candidate members of three gene families related to auxin transport. When treated with NPA or TIBA, plants cannot form the compacted heads observed in control groups.

To understand the auxin response in Chinese cabbage, we used a genome-wide analysis to characterize three PAT-related gene families, *LAX, PIN* and *PGP*. We systematically analyzed the *BrLAX, BrPIN* and *BrPGP* gene families’ chromosome distributions, gene structures, phylogenic relationships and expression profiles. Specifically, two PATIs were used to confirm the response patterns of candidate genes in leafy head formation and leaf folding. The qRT-PCR analysis showed that some of the PAT-related genes in these three families contributed to the uneven auxin distribution in outer heading leaves (HLs). Moreover, UPLC was used to identify IAA content in HLs with and without PATI treatments, further demonstrating that head formation requires distinct auxin distribution patterns in different segments. These results provide a foundation for further studies on leafy head formation and PAT in Chinese cabbage. This work offers evidence for the regulation of leafy head formation by auxin genes and may assist with progress in the gene-engineered breeding of high-yield and high-quality crops.

## Results

### Identification of *BrLAX, BrPIN* and *BrPGP*

Previous reports have identified 4 *AtAUX*/*LAX*, 8 *AtPIN* and 22 *AtPGP* genes in *Arabidopsis*. We obtained the *AUX*/*LAX, PIN* and *PGP* genes Chinese cabbage by searching against the Chinese cabbage genome via BLASTP and searching the potential sequences using an HMM search. Then, the sequences containing the *AUX*/*LAX, PIN* and *PGP* domains were searched using the program Pfam (*LAX* Pfam: PF01490; *PIN* Pfam: PF03547; *PGP* Pfam: PF00005 and PF00664). Finally, a total of 52 sequences encoding putative genes were identified in Chinese cabbage, including 10 *BrLAX*, 15 *BrPIN* and 27 *BrPGP* genes, and they were named *BrLAX1-10, BrPIN1-14* and *BrPGP1-27* based on their chromosomal locations. The detailed characteristics of these genes, including the gene names, locus IDs, ORF length, chromosomal positions, molecular weights, isoelectric points (PI) and domains, are shown in Table 1. The lengths of the putative proteins varied from 333 (*BrLAX9*) to 532 (*BrLAX2*), 341 (*BrPIN14*) to 797 (*BrPIN4*) and 1031 (*BrPGP24*) to 1415 (*BrPGP21*) amino acids. Their predicted PI values and molecular masses varied dramatically.

### Gene Structure and Chromosomal Analysis of *BrLAX, BrPIN* and *BrPGP*

**Figure 1:**
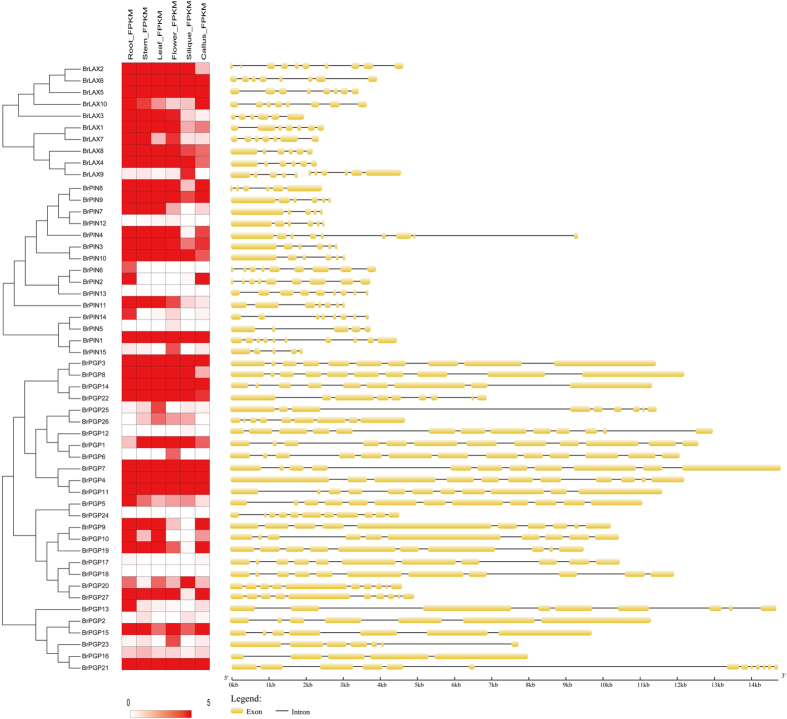
Motifs distribution and tissues-specific expression profiles of *BrLAXs, BrPINs* and *BrPGPs* in Chinese cabbage six tissues. The heat map was generated using the Cluster3.0 software. The bar on the downside represents log 2 transformed values from low to high expression.

**Figure 2:**
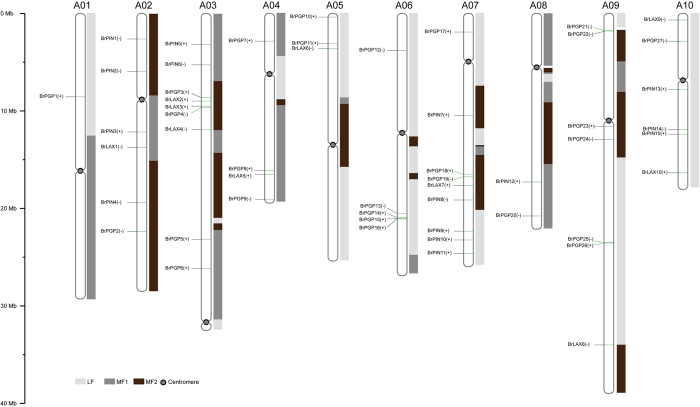
Distribution of *BrLAX, BrPIN, BrPGP* genes on 10 chromosomes and three subgenomes of Chinese cabbage. The different colored bars represent different subgenomes (LF, MF1, and MF2). The centromeres were plotted based on the Chinese cabbage genome sequencing analysis result and the size of each chromosome can be estimated by the scale on the left of the figure. The forward and reverse orientations on chromosomes were represented by positive (+), negative (−) symbols, respectively, followed each gene.

To investigate the gene structures of the *BrLAX, BrPIN* and *BrPGP* genes, the numbers and locations of introns were identified using GSDS v2.0 (http://gsds.cbi.pku.edu.cn/) (Fig. 1, Table 1). The number of introns varied from 4 to 9 in the *BrLAX* and *BrPIN* genes and from 6 to 12 in the *BrPGP* genes, representing a complex intron pattern. By analyzing the genome chromosomal location based on the *B. rapa* genome, 10 *BrLAX*s, 14 *BrPIN*s and 27 *BrPGP*s were distributed on all 10 chromosomes (Fig. 2). Briefly, most of the genes (36/51, 70.6%) were distributed on chromosomes A02, A03, A07, A09 and A10. The other genes (19/51, 29.4%) were distributed on the other chromosomes. Notably, only *PGP* genes were present on chromosome A06. In a previous study of the evolution of polyploid genomes, the *B. rapa* genome was divided into three subgroups, including the least fractionated (LF), medium fractionated (MF1) and most fractionated (MF2)^30^. In our study, most of the genes (24/51, 47.1%) belonged to the LF group, followed by 16 genes belonging to MF1 and 11 genes in MF2.

### Conserved Motifs and Phylogenetic Analysis of *BrLAX, BrPIN* and *BrPGP*

**Figure 3:**
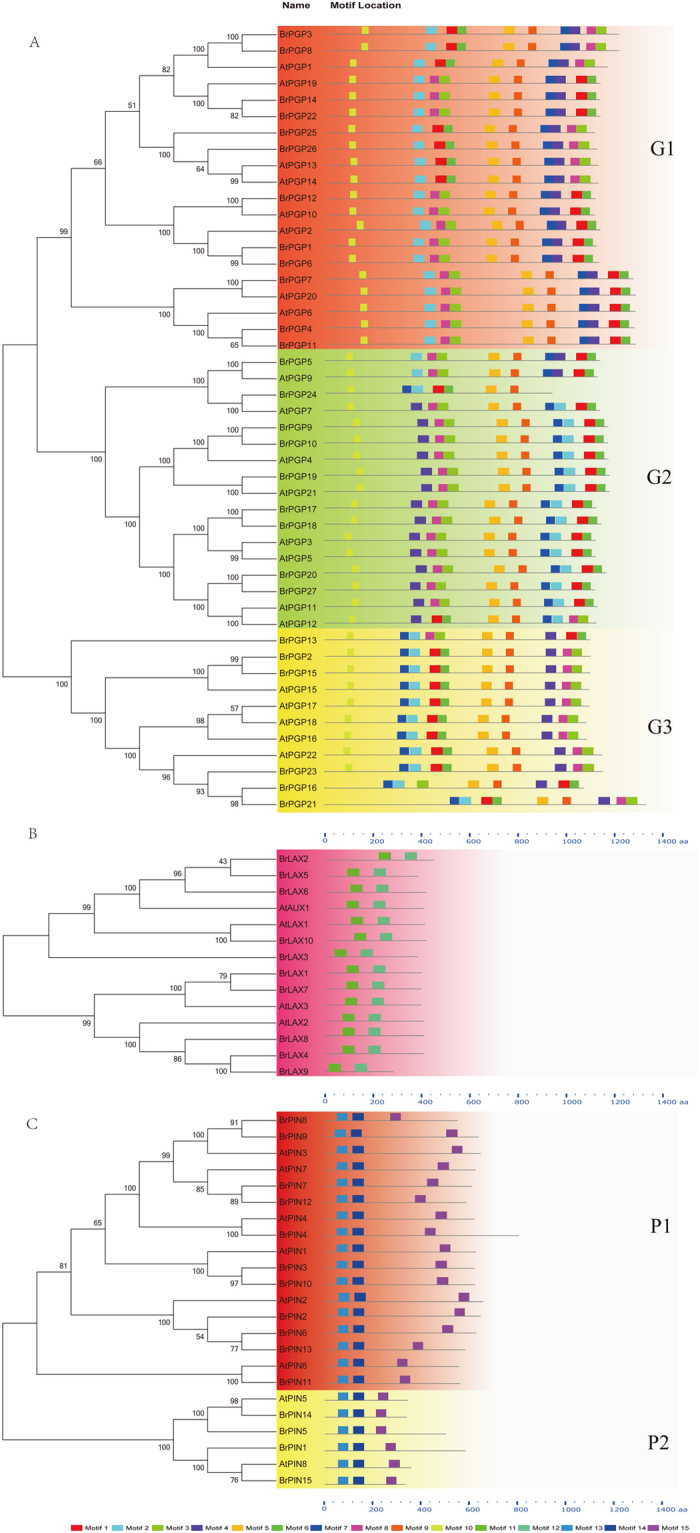
Phylogenetic relationship and conserved motifs distribution of *BrLAX, BrPIN, BrPGP* proteins. (**A**) showed 3 phylogenetic groups of each gene family evolutionary relationships of *Arabidopsis* and *brassica rapa*, which generated by MEGA5.0 with NJ method. (**B**) showed the motif structures of three gene families. The different colors LOGOs from 1 to 15 were discribed by protein sequences.

A total of 51 gene members from three gene families were identified, and the Pfam database was analyzed to locate their structural domains and conserved motifs via the MEME site. The 10 *BrLAXs* varied in length. Previous evidence showed that 4 *LAX* genes encode functional auxin influx carriers^18^. The core regions of all of the *LAX* genes showed high conservation, with 10 predicted transmembrane helices. Ten motifs were used to identify the *BrLAX, BrPIN* and *BrPGP* structures, and the motif logos are listed (Fig. 3, Fig. S1). *BrLAX* motifs 1 and 2 each contained 2 transmembrane helices. The *BrLAX* genes appear to encode transmembrane proteins, similar to *AtLAXs*, indicating that the *BrLAX* genes might perform similar biological functions in Chinese cabbage. The *PIN* family members contain a highly conservative domain structure, consisting of two hydrophobic domains divided by a hydrophilic loop containing three conserved regions (C1–C3) and two variable regions (V1 and V2)^31^. Three *PIN* motifs were located in the hydrophobic domain: motif 1 contained 2 transmembrane helices, motif 2 contained 1 transmembrane helix, and motif 3 contained 2 transmembrane helices. The multiple sequence alignment suggested that most *BrPGPs* contained two nucleotide-binding domains (NBDs) and two transmembrane domains (TMDs).

To investigate the phylogenetic relationships of *BrLAX, BrPIN* and *BrPGP* with the genes from the model plant *Arabidopsis*, a phylogenetic tree was constructed in MEGA v5.1, using the NJ method and a bootstrap value of 1,000 replicates. A total of 14 *AUX*/*LAX*, 23 *PIN*, and 49 *PGP* genes, including 4 *AtAUX*/*LAX*, 8 *AtPIN* and 22 *AtPGP*, were used to construct the phylogenetic tree. The sequences of *BrLAXs* are highly similar. The phylogenetic relationship of *PIN* is more complicated. Twenty-three *PINs* were divided into two groups (P1 and P2). Group P1 contained 17 genes, including two paralogous gene pairs (*AtPIN4*/*BrPIN4, AtPIN6*/*BrPIN11*). Compared with P1, the P2 proteins were shorter. According to the sequence similarity and phylogenetic tree topology, the 27 *BrPGP* genes were divided into 3 groups (G1–G3). Remarkably, most of the genes in the same group possessed similar gene structures. Genes in the same group that contain highly similar motif structures are likely to have similar functions.

### Expression Patterns of *BrLAX, BrPIN* and *BrPGP* Genes in Various Tissues

The gene expression profiles of the *BrLAX, BrPIN* and *BrPGP* genes in different tissues (root, stem, leaf, flower, silique, and callus) were compiled using Illumina mRNA-seq data (http://brassicadb.org/brad/genomeDominanceData.php)^32^ (Fig. 1, Table S2). A few genes (including *BrPIN5, BrPIN12, BrPIN13, BrPGP12, BrPGP17, BrPGP18*, and *BrPGP24*) did not show constitutive expression or showed relatively low levels in all six tissues, which suggested that these genes do not express or express at a specific developmental stage or under specific treatment. Most genes were highly expressed in root, with a lightly lower expression in stem and leaf, indicating that these genes may play a significant role in Chinese cabbage.

Notably, genes of the *BrLAX* family were expressed at significantly higher levels than the other two gene families, suggesting that *BrLAX* may be more important in Chinese cabbage development. Two genes, *BrPIN2* and *BrPIN6*, showed organ-specific expression in root, which was similar to the paralog *AtPIN2* identified from the phylogenetic tree^31^, suggesting that *BrPIN* and *AtPIN* may have similar biological functions. Similarly, *BrPGP9* and *BrPGP10* were strongly expressed in roots, which is consistent with the paralog *AtPGP4*^28^. *BrPGP26* showed relatively high expression in leaf, indicating that different genes might have distinct functions in maintaining plant development and morphogenesis in Chinese cabbage.

### Expression Levels of *BrLAX, BrPIN* and *BrPGP* Genes in Chinese Cabbage

**Figure 4:**
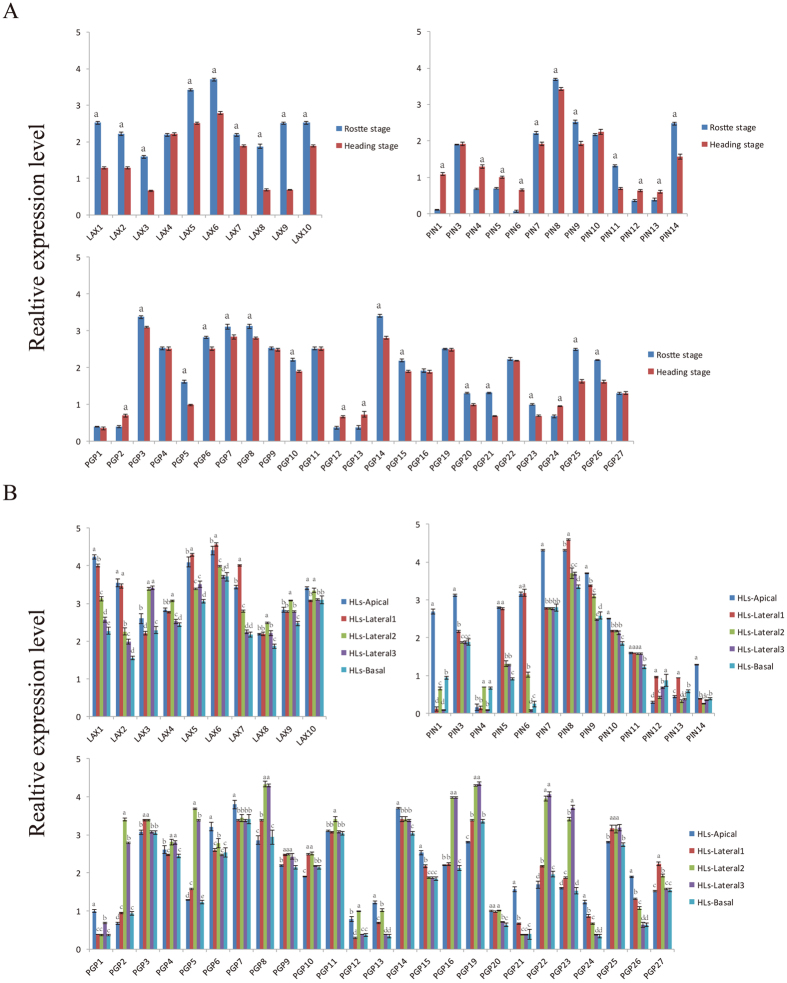
The relative expression values of 10 *BrLAX*, 15*BrPIN* and 27*BrPGP* genes in rosette leaves and heading leaves. HLs: Heading Leaves. RLs: Rosette Leaves. Error bars represent SD from three biological replicates. The different alphabetic characters in each column demonstrate significant statistical differences (p < 0.05).

Auxin is biosynthesized in meristematic tissue regions at the shoot apex and transported into different parts of the plants^5^. Thus, we propose that the extreme leaf morphology might be caused by PAT. To further identify the roles of the *BrLAX, BrPIN* and *BrPGP* genes in head formation in Chinese cabbage, the transcription of the three gene families was analyzed via quantitative real-time PCR (qRT-PCR) using the 7th rosette leaf and the 25th head leaf at the heading stage (Fig. S2). These results showed that almost all of the evaluated genes were up-regulated during the rosette stage (Fig. 4A, Table S3), the stage at which we hypothesize that the auxin transport genes, *BrLAX*s, *BrPIN*s and *BrPGP*s, began to affect the uneven auxin distribution in the rosette stage to generate the critical parameters of head formation. To identify the three gene families’ specific functions in auxin transport, HLs were divided into five parts (Apical, Lateral 1, Lateral 2, Lateral 3, and Basal) (Fig. S2). Along the vertical axis, the expression levels of most *BrLAXs* (*BrLAX1, 2, 5, 6, 7, 10*), most *BrPINs* (*BrPIN1, 3, 4, 6, 7, 8, 10, 11, 14*) and some *BrPGPs* (*BrPGP1, 6, 7, 13, 14, 15, 21, 24, 26*) in HLs-apical were higher than in the other 4 parts, while they were relatively low in the lateral and basal segments. Interestingly, we found that *BrLAX1, 2, 5*, and *7* were highly expressed in the apical regions. Along the transverse axis, some *BrPGPs* (*BrPGP2, 5, 8, 9, 10,16, 19, 22, 23, 25, 27*) and *BrPIN5, 9, 12*, and *13* showed relatively high expression in lateral parts (Fig. 4B, Table S4). Additionally, *BrPIN2* and *15* as well as *BrPGP17* and *18* did not show any expression signal, suggesting that these genes may be expressed in other organs, at a specific developmental stage or under a specific treatment.

### Most *BrLAX, BrPIN* and *BrPGP* Genes are Inhibited by NPA and TIBA

**Figure 5:**
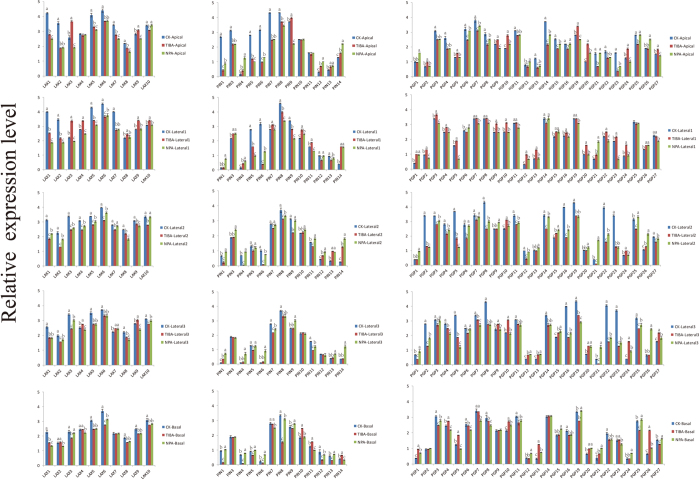
The relative expression values of 10 *BrLAX*, 15*BrPIN* and 27*BrPGP* genes treatment by NPA and TIBA. CK-Apical, CK- Lateral, CK-Basal: Different segments in heading leaves. TIBA-Apical, TIBA- Lateral, TIBA-Basal: Different segments under TIBA treatment. NPA-Apical, NPA- Lateral, NPA-Basal: Different segments under NPA treatment. The data were analyzed by three independent repeats, and standard deviations were shown with error bars. The different alphabetic characters in each column demonstrate significant statistical differences (p < 0.05).

To further investigate the functions of the three gene families in heading development, we conducted a PATI experiment using the PATIs 1-naphthylphthalamic acid (NPA) and 2,3,5-triiodobenzoic acid (TIBA). We sprayed NPA and TIBA solutions at the level of 7th rosette leaf for 30 days. Surprisingly, the plants in the open field could form tight heads, while those under the NPA or TIBA treatment failed to form any obvious head, which we hypothesized may have been because PATIs regulated some genes at the expression level. The results showed that most *BrLAX, BrPIN, BrPGP* genes were down-regulated or showed no significant change under the inhibitor treatment. For example, most *BrLAX* genes (*BrLAX1, 2, 5, 6, 7, 8, 10*) were down-regulated by PATIs, while *BrLAX3* was up-regulated in Apical and Lateral 1, and *BrLAX4* was slightly up-regulated in Lateral 1, Lateral 3 and Basal by TIBA (Fig. 5, Table S4). The expression of the *BrPIN* genes (*BrPIN3, 5, 7, 8, 9, 11*) was down-regulated by the NPA treatment. The samples treated with TIBA presented segment-specific responses; *BrPINs* were down-regulated in Apical, Lateral 2, Lateral 3, and Basal, whereas they were up-regulated or did not exhibit significant changes in Lateral 1. The *BrPGP* genes presented a similar response to the two PATIs; most exhibited down-regulation, except for *BrPGP10, 15, 20, 24, 26*, which were highly up-regulated under the two PATI treatments. Interestingly, *BrPGP5* was down-regulated by the NPA treatment but up-regulated by the TIBA treatment. Taken together, nearly all genes were down-regulated by the NPA treatment, although some genes showed tissue-specific expression. The transcriptional fluctuation of the polar auxin transport genes might be blocked by the PATIs.

### The Uneven Distribution of Auxin in Chinese Cabbage Head

**Figure 6:**
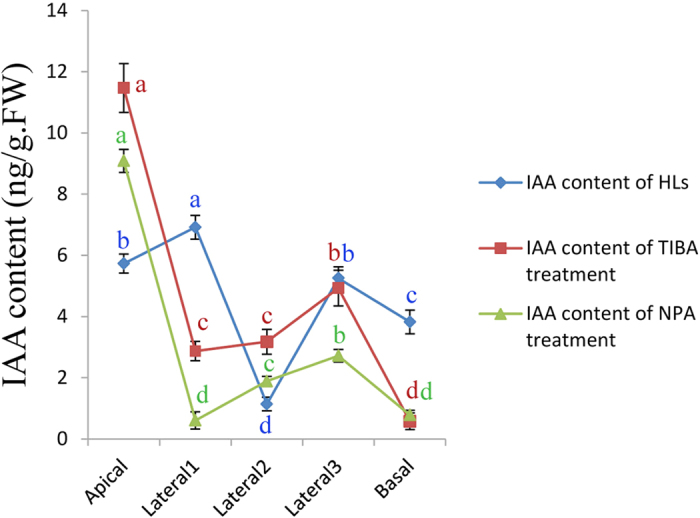
IAA contents in different treatments. The data were analyzed by three independent repeats, and standard deviations were shown with error bars. The different alphabetic characters in each column demonstrate significant statistical differences (p < 0.05).

To investigate the effect of PAT in head formation in Chinese cabbage, the IAA content of Chinese cabbage treated with TIBA and NPA and control was detected by Ultra performance liquid chromatography–tandem mass spectrometry (UPLC–MS/MS). Here, we present the hormone profile of Chinese cabbage HLs and their responses to the two PATIs in different segments of HLs (Fig. 6, Table S5). The IAA level of HLs differed significantly among the five regions; the maximum IAA content in these samples was in Lateral 1, followed by Apical, Lateral 3, Basal, and finally Lateral 2. In a previous report, TIBA and NPA were suggested to be involved in PAT^26^,^33^. Treatments with TIBA or NPA caused a similar endogenous distribution of IAA. The IAA contents in Lateral 1 and Basal were significantly lower than in the controls, perhaps due to the inhibitory effects of the PATIs. IAA rapidly accumulated in apical tissue but did not exhibit an upward curve, which might have been inhibited by the high IAA concentration. All the above findings demonstrate that PATIs can block PAT, leading to the formation of an abnormal head. The uneven distribution of auxin in the leafy head is required for formation of the leafy head.

## Discussion

Chinese cabbage is a popular and economically important vegetable. Its vegetative growth is divided into the following four stages: seedling, rosette, folding and heading^1^,^34^. The leafy head possesses leaves that are extremely incurved in the transverse and longitudinal axes, which we hypothesize might be correlated with the auxin uneven distribution. Auxins are phytohormones that regulate many developmental processes to adapt to the environment and to crop domestication^9^,^10^. IAA is the most abundant auxin in plants, and its concentration gradient is also an important auxin flow in plant development^35^. As an ancient signaling molecule, IAA promotes plant growth and development. Different IAA concentration gradients can lead to morphotype diversification. In this study, we detected the IAA contents of different segments of head and non-head leaves with PATI treatment (Fig. 6). The influx and efflux carriers of IAA regulated PAT to generate different hormone gradients, which were involved in cellular IAA transport events. Along the vertical axis of the leafy head, IAA biosynthesis in the apical region partly leads to abundance in this area, while auxin transport-associated genes respond to the auxin gradient. IAA is transported downward, and enrichment in the medial part results in a relatively high IAA content, which may lead to the vigorous outgrowth of plants’ medial tissue and to leafy head formation. Meanwhile, some auxin response genes respond to the auxin gradient and transport IAA from medial to basal, making the whole leaf erect. Along the horizontal axis, *BrPGP*s regulated the auxin distribution by transporting auxin from Lateral 1 to the margin region, which led the head leaf to bend inward. The properties of Chinese cabbage leaf folding and the incurving process appear to be physiological responses to auxin.

Head formation is an essential morphological index in Chinese cabbage. The expression levels of the *BrLAX, BrPIN* and *BrPGP* genes in HLs were lower than those in RLs, possibly indicating that the auxins were not transferred in this stage but were transferred mainly for leaf development. RLs oriented the folding inward, as a morphological marker of leaf folding. After the rosette stage, the control plants produced a normal morphology, while those with the PATI treatment did not. The responses of PAT-associated genes were then evaluated in leafy heads. The routes of PAT could be attributed to gene function in PAT. Auxin biosynthesis in the apical meristem appeared to produce a higher concentration of auxin in the Apical region (Fig. 6). Subsequently, auxin response genes conduct a dynamic distribution of auxin and the subsequent responses to leaf-folding signals. PAT genes came to play a role in transferring auxin to other segments needed for head folding (Figs 4 and 5). In the folding stage, while numerous auxins were actively transported downward along the vertical and transverse axes via PAT response factors. These results confirmed that the uneven distribution of auxin and site-specific high IAA content are indispensable in head formation.

PATI-mediated blockade of the expression of PAT-related genes was observed to potentially influence Chinese cabbage head formation. We performed PATI (NPA and TIBA) treatment to identify where and how PATIs affect PAT-regulated genes in Chinese cabbage head leaves. Many genes were down-regulated by PATIs, and some genes showed specific expression under TIBA treatment. These data indicate that the transcription of PAT genes can be blocked by PATIs, thus preventing the auxin uneven distribution required in the whole head leaves. The results were consistent with our prediction; in HLs, we found that IAA accumulated in the Apical, Lateral 1 and Basal regions, to assist with the apical closing, central excess growth, and basal straightening in HLs, while plants under PATI treatment presented abnormal morphology.

**Figure 7:**
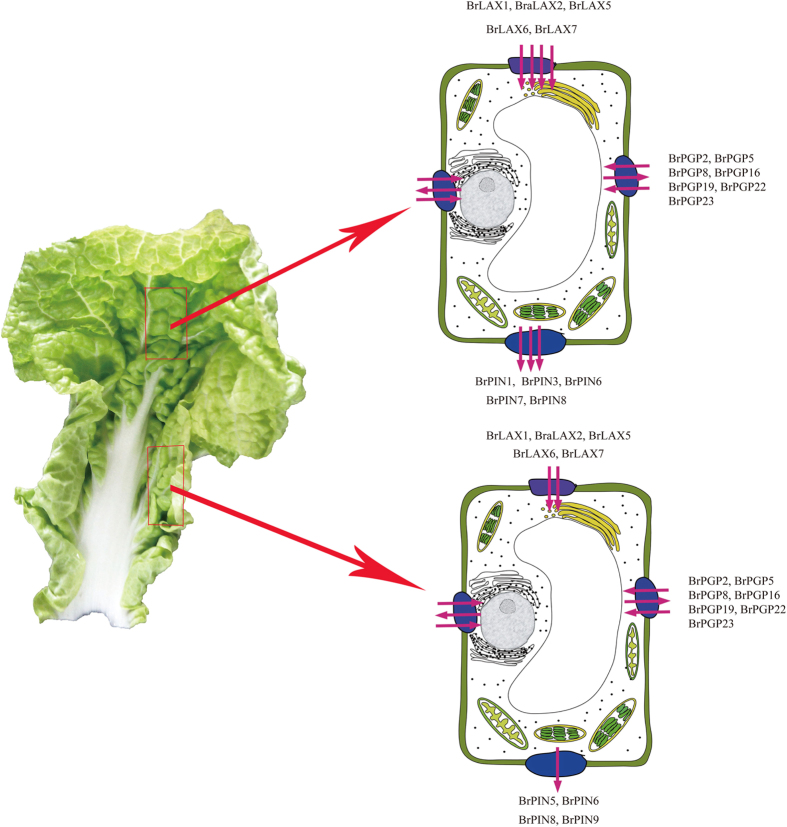
The prediction of auxin polar transport in Chinese cabbage incurved leaf. The directions of the arrows indicate auxin influx or efflux in Chinese cabbage heading leaf.

We predicted the roles and molecular mechanisms of *BrLAX, BrPIN* and *BrPGP* genes in the process of leafy head formation in Chinese cabbage (Fig. 7). Auxin was generated in meristematic tissue regions at the shoot apex and showed an uneven distribution in leaves. For HLs, the Gaussian curvature theory of Nath *et al*. explains that Lateral 1 region of the HLs grows more quickly than the margin regions^36^, and the leaf curvature induced the leafy head formation, which we predicted was caused by uneven auxin distribution in leaves. The directions and sizes of arrows represent the auxin diffusion flux and relative quantity. In a previous report, *AUX*/*LAX* was the importer of auxin into plant cells, while *PIN* was the exporter and *PGP* was a facultative transporter^37^,^38^. Uneven auxin distribution is produced via the following two pathways: passive diffusion and active transport through PAT-related proteins. In our study, we investigated the potential biological functions of *BrLAX, BrPIN* and *BrPGP* genes in the leafy heads of Chinese cabbage. Along the longitudinal axis of the Chinese cabbage leafy head, auxin plays different roles in different regions, such as apical closing, central excess growth, and basal straightening. We deduced that the auxin was transported from its site of synthesis to the basal region by PAT-related genes. In control plants, auxin influx gradually increased from the Apical region to the Basal region, which might be mediated by *BrLAXs* (*BrLAX1, 2, 5, 6, 7*) and some *BrPINs* (*BrPIN1, 3, 5, 6, 7, 8, 9*). Thus, auxin was transported from the apical region to central region and then to the basal region. For apical-to-central transport, auxin importer factors, *BrLAXs*, generate influx and accumulation of auxin in the central part, maintaining the excess growth of the leafy head, and some of the auxin was then exported to the basal region by *BrPINs* (*BrPIN1, 3, 6, 7, 8*). Meanwhile, some *BrPINs* (*BrPIN5, 6, 8, 9*) were employed to export auxin in the basal region to straighten the entire leaf. Along the transverse axis, *BrPGPs* (*BrPGP2, 5, 8, 16, 19, 22, 23*) play a crucial role in cell-to-cell PAT in order to maintain the growth of lateral regions.

Approximately 13–17 million years ago, the *Brassica* genome underwent a whole-genome triplication (WET) event^30^,^39^. The following three *Brassica* subgenomes have been demonstrated: the dominantly least fractionated (LF) subgenome and the remaining two more-fractionated subgenomes (MF1 and MF2). The ‘Triangle of U’ model was established to encompass three diploid species, *B. rapa* (A genome), *B. Nigra* (B genome), and *B. Oleracea* (C genome), and three amphidiploid species, *B. juncea* (AB genomes), *B. napus* (AC genomes) and *B. carinata* (BC genomes), by pairwise hybridization. In previous studies, three gene families, including *AUX*/*LAX, PIN*, and *PGP*, were identified as related to auxin polar transport and functions in various biological processes^15^,^16^,^18^,^19^,^20^,^25^,^40^. We investigated the phylogenetic relationships of these auxin transport proteins in different species and constructed a phylogenetic tree of *B. rapa* and *Arabidopsis*. Cheng *et al*. reported that Chinese cabbage (*B. rapa*) and cabbage (*B. oleracea*) possess similar leaf heading, which might be attributable to various processes, such as auxin-mediated signaling. Auxin-mediated signaling pathways were hypothesized to regulate leaf adaxial-abaxial patterning in leaf-heading morphotypes of both *B. rapa* and *B. oleracea*. Several essential genes in leafy head formation (*BRX*s, *ARF*s, *KAN*s and *AXR*s) were identified^39^, and they have been shown to interact with PATs to create auxin gradients^41^,^42^,^43^. The temporal differences in expression of PINs involved in the Aux/IAA-ARF (indole-3-acetic acid-auxin response factor) signaling pathway result in the auxin-mediated regulation of PAT^44^. In this study, we found that most of the auxin genes were expressed in multiple leaf segments, suggesting that they might have different functions in Chinese cabbage development. Thus, auxin, the main phytohormone, induced a series of complicated biochemical mechanisms of leafy head formation that could elucidate the relationship between PAT genes and the establishment of morphogenesis, which might lead to better understanding of leafy head formation and organogenesis in other species. Here, we hypothesize that the similar leafy head phenotypes in Chinese cabbage and cabbage might be due to the same form of uneven auxin distribution, which requires further study.

In summary, we first propose a model of auxin polar transport in Chinese cabbage head leaves and reveal the auxin flow or accumulation in the whole head leaf and the uneven auxin distribution associated with leafy head formation in Chinese cabbage (Fig. 7). We hypothesize that some genes of the *BrLAX, BrPIN* and *BrPGP* families play essential roles in the uneven auxin distribution. We show the auxin biosynthesis in meristematic tissue regions at the shoot apex and the downward auxin flow to modify the lateral outgrowth. At the cell level, the *BrLAX* auxin influx factors mainly regulated the auxin response and the subsequent downward polar auxin transport. *BrPIN*s participate in the auxin transport out of cells, and auxin accumulates in medial regions to ensure their outgrowth. *BrPGP*s are auxin response factors focused on PAT during development along the transverse axis, producing an inward bend to head leaves. Those results also indicated that the *BrLAX, BrPIN* and *BrPGP* genes may be involved in the PAT during leafy head development, which merits further functional exploration in the future.

## Materials and Methods

### Plant Materials and Treatment of the Foliage with Chemicals

Seeds of the inbred line ‘chiifu-401-42’ were employed and germinated on moisture-absorbent papers, grown at 25 °C for 3 d and then transplanted to a climate-controlled chamber with 16 h light/18 dark cycles. The seedlings with growth substrate came from Nanjing Agriculture University at the normal sowing time. After cultivation for 14 days, for the rosette leaves (RLs), entire leaves were harvested. The RLs were sprayed with solutions of 10 mM NPA or 50 mM TIBA for 30 days. The NPA and TIBA were purchased from Aladdin (Aladdin Industrial Corporation, Shanghai, China). For each treatment condition, three biological replicates were established to reduce the error rate. The mixed HLs (HL-mix), HL-Apical, HL-Lateral 1, HL-Lateral 2, HL-Lateral 3, and HL-Basal materials were harvested at the early folding stage, using 25 expanded leaves for expression analysis and phytohormone assays (Fig. S2).

### Identification of *AUX*/*LAX, PIN* and *PGP* Genes in Chinese Cabbage

To identify the *AUX*/*LAX, PIN*, and *PGP* genes in Chinese cabbage, the genome sequences of Chinese cabbage were retrieved from BRAD (http://brassicadb.org/brad/). Gene sequences of *AtAUX*/*LAX, AtPIN* and *AtPGP* genes were obtained from TAIR (http://arabidopsis.org/index.jsp)^45^, and these sequences served as seeds to obtain the target sequence in the *B. rapa* genome using BLAST v2.2.27+. Then, HMMER v3.0 (http://hmmer.janelia.org/) was used to search for all candidate genes in the entire genome sequences, based the specific HMM profile. A total of 52 genes were obtained after identification using BLASTP (E-value ≤ 1e-20), and the Pfam (E-value ≤ 1e-4) database (http://pfam.sanger.ac.uk/) was used to identify the genome assemblies of *Arabidopsis* (http://arabidopsis.org/index.jsp) and Chinese cabbage (http://brassicadb.org/brad/). To investigate the characteristics of the putative *BrLAX, BrPIN* and *BrPGP* proteins, Pepstats (http://www.ebi.ac.uk/Tools/seqstats/emboss_pepstats/) was used to identify their molecular weights (MW) and isoelectric points (pI), and MEME v. 4.10.1 (http://meme-suite.org/tools/meme)^46^ was used to identify the conserved motifs in *BrLAX, BrPIN* and *BrPGP* genes.

### Phylogenetic Analysis of *BrLAX, BrPIN* and *BrPGP* Genes

The protein sequences were screened against the Pfam database (http://pfam.sanger.ac.uk/) in order to further confirm the putative domains, which were aligned using ClustalX 2.0 with the default parameters^47^. MEGA v5.1 was used to construct Neighbor-Joining (NJ) phylogenetic trees using Chinese cabbage and *Arabidopsis* protein domain sequences with a bootstrap of 1,000 replicates.

### Expression Profiling Analysis of *BrLAX, BrPIN* and *BrPGP* Genes

To characterize the expression patterns of the *BrLAX, BrPIN* and *BrPGP* genes, we used Illumina RNA-Seq data, as previously described^48^. The expression level was calculated as fragments per kilobase of exon model per million mapped (FPKM) values. Gene expression patterns were analyzed using Cluster v3.0 (http://bonsai.hgc.jp/~mdehoon/software/cluster/) and were calculated as FPKM values. The heat maps of hierarchical clustering were established using Tree View v.3.0 (http://jtreeview.sourceforge.net/) based on the log2-converted FPKM values.

### RNA Extraction and Quantitative Real Time-PCR (qRT-PCR) Analysis

Total RNA was isolated from different parts of the leaves using an RNA extraction kit (TaKaRa RNAiso Reagent, Takara, Dalian, China) according to the manufacturer’s protocols and was treated with DNase I (TaKaRa). Gel electrophoresis was used to assess RNA quality and quantity. Subsequently, 2 μg of total RNA was used to synthesize first-strand cDNA using the PrimeScript 1st Strand cDNA Synthesis Kit (Takara, Dalian China). Three biological and three technical replicates were used to reduce the error rate. These specific primers were designed using Beacon Designer v7.9 (Table S1). The *Actin* gene (*Bra028615*) was used as an internal control^49^. Each reaction contained diluted cDNA, specific primers and the SYBR® Premix ExTaq™ (Takara, Dalian, China), to identify the gene expression, according to the product manual for the Step One Plus Real-Time PCR system (Applied Biosystems) with the following conditions: 94 °C for 30 s; followed by 40 cycles at 94 °C for 10 s, 58 °C for 30 s; the melting curve (61 cycles at 65 °C for 10 s) was performed to check specific amplification.

### Quantification of Phytohormone in Different Parts of the Heading Stage

Total IAA was isolated and quantified using UPLC, as previously reported by Novak *et al*.^50^. All experiments were conducted using three repeats. The heading leaves were separated into five parts in liquid nitrogen and then extracted with 80% (v/v) methanol at 4 °C in the dark for 12 h. Next, the supernatant was collected after centrifugation for 10 min at 10,000 rpm. The residue was extracted twice as described above, and then the supernatants were purified on C18 Sep-Pak cartridges and freeze-dried before being dissolved with pure methanol. Finally, the supernatants were filtered through a 0.22 μm PTFE filter. The total IAA concentrates were analyzed via UPLC on an Agilent 1290 Infinity. Analytical reagent grade IAA was purchased from Aladdin (Aladdin Industrial Corporation, Shanghai, China). The absorption area value of the IAA was calculated manually.

### Data Analysis

The relative gene expression level was calculated using the comparative Ct method. RNA quantification related to the actin gene expression level was performed using the 2^−∆∆CT^ method, as reported previously^51^. The data were analyzed using SPSS software (SPSS version 19.0, SPSS, Chicago, IL, USA), using descriptive statistical tests; one-way analysis of variance was used to evaluate the differences among categories. Statistical significance was established at 0.05.

## Additional Information

**How to cite this article**: Gao, L.-w. *et al*. Genome-wide analysis of auxin transport genes identifies the hormone responsive patterns associated with leafy head formation in Chinese cabbage. *Sci. Rep.* **7**, 42229; doi: 10.1038/srep42229 (2017).

**Publisher's note:** Springer Nature remains neutral with regard to jurisdictional claims in published maps and institutional affiliations.

## Supplementary Material

Supplemental Figure

Supplementary Table S1

Supplementary Table S2

Supplementary Table S3

Supplementary Table S4

Supplementary Table S5

## Figures and Tables

**Table 1:** Details of auxin transport genes in Chinese cabbage.

Gene	B. rapa Gene ID	*A. thaliana* ID	Chromosome location (bp)	aa length (bp)	No. of introns	PI	Molecular Weight(Da)
*BrLAX1*	Bra008325	AT1G77690	A02:14725754–14728210	1419	6	4.7852	119672.77
*BrLAX2*	Bra000039	AT2G38120	A03:8969103–8973665	1599	9	4.747	133793.03
*BrLAX3*	Bra000160	AT2G38120	A03:9634033–9635966	1365	5	4.9753	114881.36
*BrLAX4*	Bra000584	AT2G21050	A03:11901921–11904188	1452	5	4.7624	122297.26
*BrLAX5*	Bra017136	AT2G38120	A04:16416566–16420434	1368	7	4.7644	115542.26
*BrLAX6*	Bra005136	AT2G38120	A05:3621967–3625352	1482	7	4.75	125234.98
*BrLAX7*	Bra003674	AT1G77690	A07:14390353–14392671	1416	6	4.8035	118872.16
*BrLAX8*	Bra031158	AT2G21050	A09:32252231–32254398	1452	5	4.7592	121956.97
*BrLAX9*	Bra015546	AT2G21050	A10:1136558–1138329	1002	4	4.8244	85540.75
*BrLAX10*	Bra009636	AT5G01240	A10:17540102–17543701	1488	7	4.7488	124948.61
*BrPIN1*	Bra023503	AT5G15100	A02:3652217–3656672	1794	9	4.7331	150254.44
*BrPIN2*	Bra035648	AT5G57090	A02:6964762–6968505	1986	8	4.7022	164375.95
*BrPIN3*	Bra008105	AT1G73590	A02:13147483–13150339	1851	5	4.6798	155118.8
*BrPIN4*	Bra026669	AT2G01420	A02:21301646–21310956	2394	8	4.6151	201048.87
*BrPIN5*	Bra006361	AT5G16530	A03:3166219–3169969	1542	4	4.7803	129131.69
*BrPIN6*	Bra006834	AT5G57090	A03:5266421–5270315	1929	8	4.6998	159708.46
*BrPIN7*	Bra012358	AT1G23080	A07:8240057–8242519	1812	4	4.6926	151523.13
*BrPIN8*	Bra003938	AT1G70940	A07:15855206–15857696	1641	5	4.7097	137290.75
*BrPIN9*	Bra016173	AT1G70940	A07:19048198–19050882	1899	5	4.6673	159650.56
*BrPIN10*	Bra015983	AT1G73590	A07:19950892–19953957	1857	5	4.6772	155481.21
*BrPIN11*	Bra015694	AT1G77110	A07:21325361–21328418	1725	6	4.7195	145180.14
*BrPIN12*	Bra016366	AT1G23080	A08:18065553–18068065	1755	5	4.7107	147297.11
*BrPIN13*	Bra002763	AT5G57090	A10:7747823–7751512	1788	8	4.7238	148933.04
*BrPIN14*	Bra008615	AT5G16530	A10:13100022–13102078	1047	7	4.8676	87432.14
*BrPIN15*	Bra008722	AT5G15100	A10:13574222–13576153	1026	4	4.8822	85709.37
*BrPGP1*	Bra013936	AT4G25960	A01:8540846–8546690	3705	11	4.5678	305342.58
*BrPGP2*	Bra033043	AT3G28345	A02:21713331–21718585	3735	6	4.5994	305068.65
*BrPGP3*	Bra023087	AT2G36910	A03:8624046–8629351	4020	9	4.5341	332341.52
*BrPGP4*	Bra000136	AT2G39480	A03:9481070–9486731	4212	10	4.5557	348938.17
*BrPGP5*	Bra012621	AT4G18050	A03:23170309–23175454	3744	11	4.575	306143.38
*BrPGP6*	Bra019135	AT4G25960	A03:26140733–26146345	3726	12	4.5692	307511.73
*BrPGP7*	Bra014756	AT3G55320	A04:2832718–2839592	4203	10	4.5458	348403.64
*BrPGP8*	Bra017216	AT2G36910	A04:15986960–15992620	4017	9	4.5303	332070.52
*BrPGP9*	Bra040475	AT2G47000	A04:18946035–18950787	3864	9	4.5869	315564.33
*BrPGP10*	Bra004484	AT2G47000	A05:370135–375843	3855	9	4.5917	314910.05
*BrPGP11*	Bra005036	AT2G39480	A05:3097621–3103960	4227	10	4.5448	349645.14
*BrPGP12*	Bra019907	AT1G10680	A06:3774311–3781400	3678	12	4.5692	303663.58
*BrPGP13*	Bra025425	AT3G28345	A06:21585065–21593092	3726	8	4.5857	307276.3
*BrPGP14*	Bra025359	AT3G28860	A06:22003874–22010062	3759	8	4.5648	310998.43
*BrPGP15*	Bra025326	AT3G28345	A06:22155393–22160709	3735	6	4.5857	305512.59
*BrPGP16*	Bra025331	AT3G28415	A06:22120036–22124400	3420	4	4.614	280349.15
*BrPGP17*	Bra002094	AT4G01830	A10:11533544–11539264	3696	10	4.6243	301021.24
*BrPGP18*	Bra003445	AT4G01830	A07:13221037–13227556	3765	10	4.6211	306744.39
*BrPGP19*	Bra003490	AT3G62150	A07:13457705–13462900	3879	9	4.6001	315685.88
*BrPGP20*	Bra030503	AT1G02520	A08:21539124–21543766	3831	9	4.5854	311476.41
*BrPGP21*	Bra039055	AT3G28415	A09:1364841–1379565	4248	12	4.5607	347908.25
*BrPGP22*	Bra039042	AT3G28860	A09:1451543–1458438	3759	8	4.5767	310398.56
*BrABBC23*	Bra027534	AT3G28415	A09:13505446–13513218	3675	6	4.5924	300859.02
*BrPGP24*	Bra017539	AT5G46540	A09:16196016–16200573	3096	10	4.6403	253321.15
*BrPGP25*	Bra032864	AT1G28010	A09:12328042–12339538	3687	8	4.5972	303846.58
*BrPGP26*	Bra032856	AT1G27940	A09:12379011–12383707	3720	8	4.5881	306865.56
*BrPGP27*	Bra033331	AT1G02520	A10:4205096–4210047	3801	9	4.5773	309226.75
