# Supplementary material for: Genome-wide analysis of auxin transport genes identifies the hormone responsive patterns associated with leafy head formation in Chinese cabbage
Source: Sci Rep. 2017 Feb 7;7:42229. doi: 10.1038/srep42229 (PMC5294403; doi:10.1038/srep42229)
Supplement: Supplemental Figure [file srep42229-s1.pdf]

Genome-wide analysis of auxin transport genes identifies the hormone responsive patterns associated with leafy head formation in Chinese cabbage

Li-wei Gao[a,1], Shan-wu Lyu[a,1], Jun Tang[b], Dao-yun Zhou[a], Guusje Bonnema[c] Dong Xiao[a], Xi-lin Hou[a], Chang-wei Zhang[a*]

[a]State Key Laboratory of Crop Genetics and Germplasm Enhancement/Key Laboratory of Biology and Germplasm Enhancement of Horticulture Crops in East China, Ministry of Agriculture, Nanjing Agricultural University, Nanjing, 210095, PR China

[b]Institute of Horticulture, Jiangsu Academy of Agricultural Sciences, Nanjing, PR China

[c]Wageningen UR Plant Breeding, Wageningen University and Research Centre, Wageningen, the Netherlands

[*]**Corresponding author:**

E-mail: changweizh@njau.edu.cn (C. Z) Telephone: +86-25-84395332 Fax:

+86-25-84395266

[1] These authors contributed equally to this work.

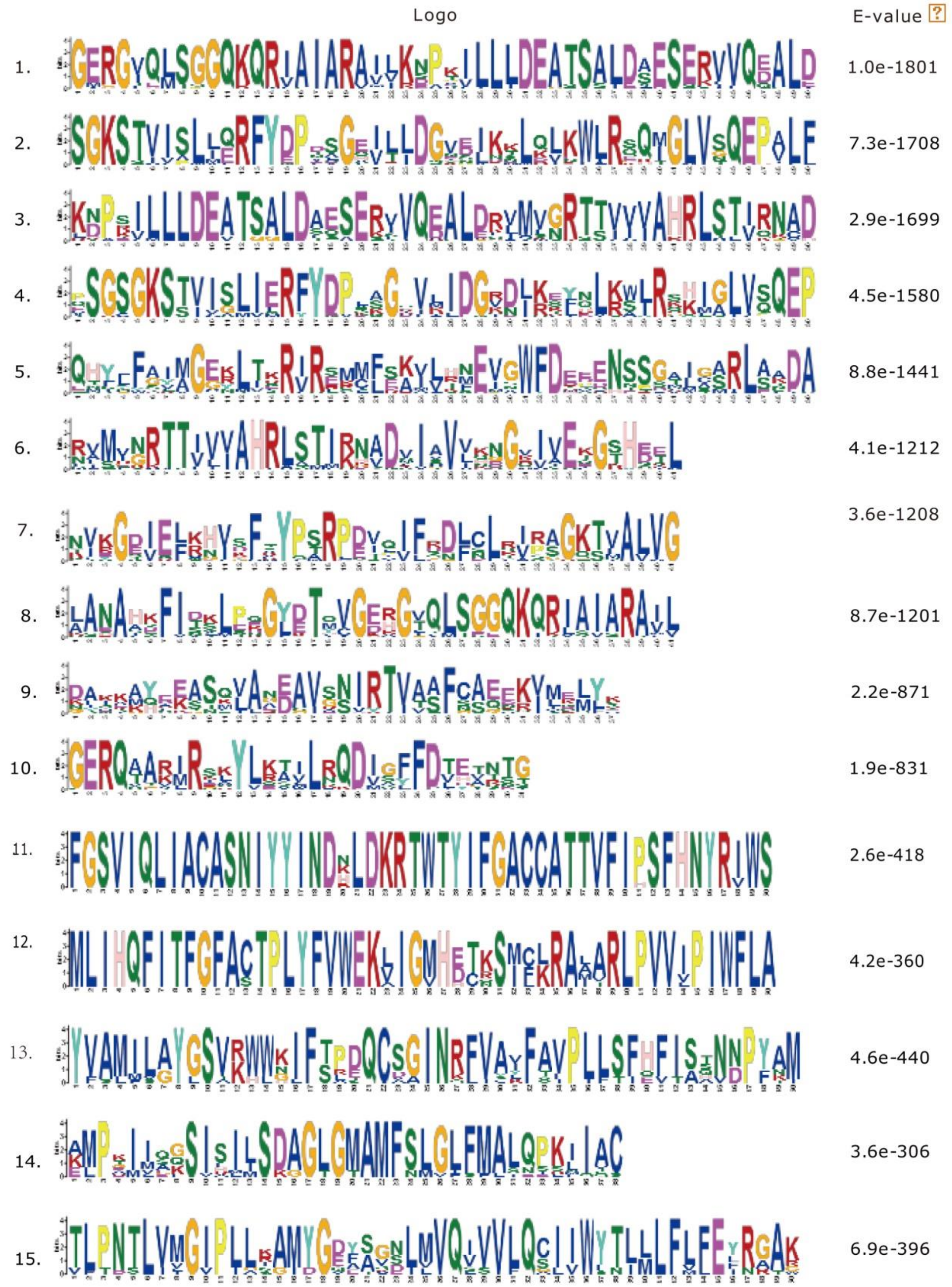

Figure S1. 15 conserved motif LOGO of 10 *BrLAXs*, 15 *BrPINs* and 27 *BrPGPs* in Chinese cabbage.

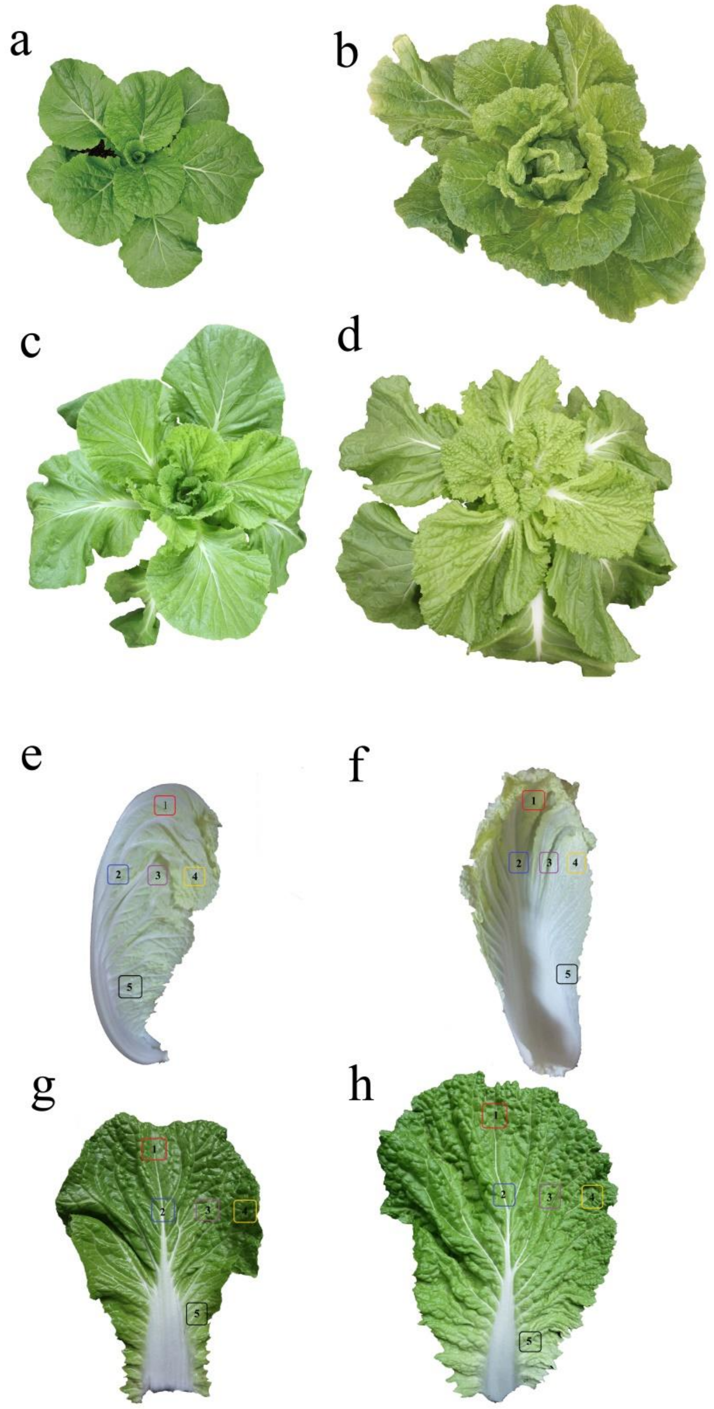

Figure S2. Phenotypes of rosette stage (a), heading stage (b), NPA treatment (c), TIBA treatment (d) and sample regions (e, f, g, h). 1: Apical regions, 2: Lateral 1 regions, 3: Lateral 2 regions, 4: Lateral 3 regions, 5: Basal regions.
